# Clinical, histological, and genetic characterization of *PYROXD1*-related myopathy

**DOI:** 10.1186/s40478-019-0781-8

**Published:** 2019-08-27

**Authors:** Xavière Lornage, Vanessa Schartner, Inès Balbueno, Valérie Biancalana, Tracey Willis, Andoni Echaniz-Laguna, Sophie Scheidecker, Ros Quinlivan, Michel Fardeau, Edoardo Malfatti, Béatrice Lannes, Caroline Sewry, Norma B. Romero, Jocelyn Laporte, Johann Böhm

**Affiliations:** 1 Institut de Génétique et de Biologie Moléculaire et Cellulaire (IGBMC), 1 rue Laurent Fries, 67404 Illkirch, France; 2 Inserm U1258, Illkirch, France; 3 CNRS UMR7104, Illkirch, France; 4 Strasbourg University, Illkirch, France; 5 Laboratoire de Diagnostic Génétique, Faculté de Médecine, CHRU, Strasbourg, France; 6 Wolfson Centre of Inherited Neuromuscular Disorders, RJAH Orthopaedic Hospital, Oswestry, UK; 7 Department of Neurology, APHP, CHU de Bicêtre, Le Kremlin Bicêtre, France; 8 French National Reference Center for Rare Neuropathies (NNERF), Le Kremlin Bicêtre, France; 9 Inserm U1195 & Paris-Sud University, Le Kremlin Bicêtre, France; 10 Laboratoire de Génétique Médicale, Strasbourg University, Strasbourg, France; 11 MRC Centre for Neuromuscular Diseases, National Hospital for Neurology and Neurosurgery, London, UK; 12 Université Sorbonne, UPMC Paris 06 University, Inserm UMRS974, CNRS FRE3617, Center for Research in Myology, GH Pitié-Salpêtrière, Paris, France; 13 Centre de Référence de Pathologie Neuromusculaire Paris-Est, Institut de Myologie, GHU Pitié-Salpêtrière, Assistance Publique-Hôpitaux de Paris, Paris, France; 14 Neuromuscular Morphology Unit, Myology Institute, GHU Pitié-Salpêtrière, Paris, France; 15 Service Neurologie Médicale, Centre de Référence Maladies Neuromusculaire Paris-Nord, CHU Raymond-Poincaré, Garches, U1179 UVSQ-INSERM Handicap Neuromusculaire: Physiologie, Biothérapie et Pharmacologie appliquées, UFR des sciences de la santé Simone Veil, Université Versailles-Saint-Quentin-en-Yvelines, Montigny-le-Bretonneux, France; 16 Department of Pathology, Strasbourg University Hospital, Strasbourg, France; 17 Dubowitz Neuromuscular Centre, UCL Institute for Child Health and Great Ormond Street Hospital, London, UK

**Keywords:** PYROXD1, Oxidoreductase, Congenital myopathy, LGMD, Myofibrillar inclusions

## Abstract

Recessive mutations in *PYROXD1*, encoding an oxidoreductase, were recently reported in families with congenital myopathy or limb-girdle muscular dystrophy. Here we describe three novel *PYROXD1* families at the clinical, histological, and genetic level. Histological analyses on muscle biopsies from all families revealed fiber size variability, endomysial fibrosis, and muscle fibers with multiple internal nuclei and cores. Further characterization of the structural muscle defects uncovered aggregations of myofibrillar proteins, and provided evidence for enhanced oxidative stress. Sequencing identified homozygous or compound heterozygous *PYROXD1* mutations including the first deep intronic mutation reinforcing a cryptic donor splice site and resulting in mRNA instability through exonisation of an intronic segment. Overall, this work expands the *PYROXD1* mutation spectrum, defines and specifies the histopathological hallmarks of the disorder, and indicates that oxidative stress contributes to the pathomechanism. Comparison of all new and published cases uncovered a genotype/phenotype correlation with a more severe and early-onset phenotypic presentation of patients harboring splice mutations resulting in reduced PYROXD1 protein levels compared with patients carrying missense mutations.

## Introduction

Myopathies are clinically and genetically heterogeneous and can be sub-classified based on the clinical presentation of the patients and notably on the presence of specific histological anomalies on muscle biopsies [5, 8]. Recently, *PYROXD1* mutations were described in patients with slowly progressive congenital myopathy, and analysis of the muscle biopsies revealed multiple internal nuclei and cores, as well as myofibrillar inclusions [6]. Additional *PYROXD1* cases were reported with childhood or adult-onset limb-girdle muscular dystrophy (LGMD) [9, 10]. PYROXD1 (pyridine nucleotide-disulfide oxidoreductase domain-containing protein 1) is expressed in a multitude of tissues, has a nuclear and cytosolic localization in skeletal muscle, and acts as an oxidoreductase potentially implicated in energy metabolism [6, 9]. *Pyroxd1* downregulation in murine C2C12 myoblasts impaired cellular proliferation, migration, and differentiation, and knockdown of the drosophila orthologue CG10721 is lethal, demonstrating that PYROXD1 is essential for normal development [9].

In total, nine families with recessive *PYROXD1* mutations have been reported to date [6, 9, 10]. The identified mutations were evenly distributed over the gene and encompassed essential splice site mutations of in-frame exons, a 4-nucleotide insertion in the penultimate exon, and missense mutations affecting highly conserved amino acids. The most common p.(Asn155Ser) mutation was found in five families at the homozygous state and in three further families at the heterozygous state in combination with another mutation. Here we report additional patients from three unrelated families harboring three known and one new *PYROXD1* mutation. We describe for the first time a deep intronic mutation and thereby highlight RNA sequencing as a method to diagnose *PYROXD1* cases. Investigations on the patient biopsies revealed fibers with foetal myosin and increased oxidative stress markers. We also compared all published and new *PYROXD1* patients and provide an overview on the clinical, histological and genetic spectrum of *PYROXD1*-related myopathy and draw a genotype/phenotype correlation.

## Materials and methods

### Patients

Sample collection was performed with written informed consent from the patients according to the declaration of Helsinki and its later amendments. DNA storage and usage was IRB-approved (DC-2012-1693).

### Morphological analyses

Patient P1 from Family 1 underwent open muscle biopsy at the age of 6, P2 (Family 2) underwent two open muscle biopsies at 29 and 66 years of age, and P3 (Family 3) underwent open muscle biopsy at the age of 9. For histological and histochemical analyses, transverse 10 μm cryostat muscle sections were stained with Haematoxylin & Eosin (H&E), Nicotinamide adenosine dinucleotide-tetrazolium reductase (NADH-TR), modified Gomori Trichome (mGT), and cytochrome c oxidase (COX). For electron microscopy, muscle sections were fixed, post-fixed, and dehydrated according to standard procedures, and embedded in epon resin. For immunohistochemistry, following primary and secondary antibodies were used: mouse anti-desmin (D33, Abcam, Cambridge, UK), mouse anti-myotilin (Novocastra, Newcastle upon Tyne, UK), mouse anti-alpha B crystallin (1B6.1-3G4, Abcam), mouse anti-p62 (D-3, Santa Cruz Biotechnology, Dallas, USA, mouse anti-foetal myosin (Novocastra), and appropriate secondary antibodies (Alexa Fluor, Invitrogen, Carlsbad, CA, USA).

### Molecular diagnosis

P1 (Family 1) was sequenced for a targeted panel of 210 neuromuscular disorders genes (MYOdiagHTS) on a NextSeq550 (Illumina), P2 (Family 2) was exome-sequenced with the SureSelect Human all Exon 50 Mb capture library v5 (Agilent, Santa Clara, USA) followed by paired-end sequencing on an Illumina HiSeq2500 (Illumina, San Diego, USA), and P3 (Family 3) was directly Sanger-sequenced for all coding exons and the adjacent splice-relevant regions of *PYROXD1*. Confirmation of variants and segregation was performed by Sanger sequencing for all families. The mutations were numbered according to GenBank NM_024854.4 and NP_079130.2 with + 1 corresponding to the A of the ATG translation initiation codon.

### RNA analyses

Skeletal muscle RNA from P3 (Family 3) and an age-matched control were extracted from frozen muscle using the Precellys 24 homogenizer (Bertin Technologies, Montigny-le-Bretonneux, France). Relative expression of *PYROXD1* was measured with the SYBR Green PCR Master Mix (Qiagen, Hilden, Germany) on a LightCycler 480 Real-Time PCR System (Roche, Basel, Switzerland) using human *PYROXD1-* and *HPRT1*-specific primer sets. For cDNA analysis, the RNA was reverse transcribed using the SuperScript® III kit (Invitrogen).

### Western blot

Total muscle lysates were prepared in a buffer containing 50 mM Tris, 100 mM NaCl, 1 mM EGTA, 0.5% NP-40, 0.5% Triton-X100, 0.1% SDS, 1 mM DTT, 1 mM PMSF, and a mix of protease inhibitor (Complete EDTA-free, Roche, Basel, Switzerland), and 50 μg of protein extracts were loaded on a 10% SDS-Page gel. The following primary and secondary antibodies were used: sc-133,245 mouse anti-Glutathione Reductase (Santa Cruz Biotechnology), rabbit anti-HSP70 (4872S, Cell Signaling Technology, Danvers, USA) mouse anti-GAPDH (MAB374, Millipore, Burlington, USA), and horseradish peroxidase (Jackson immunoresearch Europe, Cambridgeshire, UK). Membranes were revealed with the Supersignal west pico kit (ThermoFisher Scientific), and immunoblots were visualized on an Amersham Imager 600 (GE Healthcare Life Sciences, Chicago, USA). Quantifications of glutathione reductase and HSP70 reflect a single experiment.

## Results

### Clinical reports

The patients described in this study belong to three unrelated families and presented with an early-onset and progressive muscle disorder. The clinical and histological features are summarized in Table 1 and compared with all previously reported *PYROXD1* families.

**Table 1:** Clinical, genetic, and histological features of patients with *PYROXD1* mutations. All families have been numbered according to the mutation position. Homozygous mutations are highlighted in bold

Family	Patient	Mutation	Onset	Muscle weakness	Muscle histology	Nasal speech	Scoliosis	Respiration	Other features	Reference
1	P1	c.285 + 1G > A c.464A > G p.(Asn155Ser)	Neonatal	Axial, wheel-chair-bound since age 12	Internal nuclei, cores, fiber size variability, fibrosis	No	Yes	NIV and oxygen therapy since age 14	Joint hypermobility	This study
2	C.II.1	c.414 + 1G > A c.464A > G p.(Asn155Ser)	Neonatal	Axial, upper and lower limbs, facial weakness	NA	Yes	Yes	Normal	Joint hypermobility, contractures, rigid spine, high-arched palate	O’Grady et al., 2016 [6]
	C.II.2		Childhood	Axial, upper and lower limbs, facial weakness	Internal nuclei, cores, myofibrillar inclusions, sarcomeric disorganization	Yes	No	Normal	Joint hypermobility, high-arched palate	
3	P2	**c.464A > G p.(Asn155Ser)**	Childhood	Axial, upper and lower limbs	Internal nuclei, cores, myofibrillar inclusions		No	VC 68%	–	This study
4	B.II.2	**c.464A > G p.(Asn155Ser)**	Childhood	Proximal and axial, upper and lower limbs, facial weakness	NA	Yes	No	Normal	–	O’Grady et al., 2016 [6]
	B.II.3		Childhood	Proximal and axial, upper and lower limbs, facial weakness	Internal nuclei, cores, myofibrillar inclusions, sarcomeric disorganization, rods	Yes	No	Abnormal	Ptosis, retrognathia	
5	D.II.1	**c.464A > G p.(Asn155Ser)**	Childhood	Proximal and axial, upper and lower limbs, facial weakness	NA	Yes	No	Normal	Ptosis, high-arched palate	O’Grady et al., 2016 [6]
	D.II.3		Childhood	Proximal and axial, upper and lower limbs, facial weakness	Internal nuclei, cores	Yes	No	Normal	Ptosis, high-arched palate	
6	1	**c.464A > G p.(Asn155Ser)**	Childhood	Proximal, lower limbs, wheelchair-bound since age 37	NA	No	No	Normal	–	Saha et al., 2018 [9]
7	P2	**c.464A > G p.(Asn155Ser)**	Childhood	Proximal, upper and lower limbs, requires cane since age 54, facial weakness	Internal nuclei, fiber size variability	No	No	VC 40%	Ptosis, kyphosis	Sainio et al., 2019 [10]
8	P3	**c.464A > G p.(Asn155Ser)**	Adulthood	Proximal and axial, upper and lower limbs, requires cane since age 70	NA	No	No	VC 67%	–	Sainio et al., 2019 [10]
	P4		Adulthood	Proximal and axial, upper and lower limbs, wheelchair-bound since age 66	Dystrophic features, myofibrillar inclusions	No	No	VC 30%	–	
9	P1	c.464A > G p.(Asn155Ser) c.1061A > G p.(Tyr354Cys)	Adulthood	Proximal, upper and lower limbs	Internal nuclei, fiber size variability	No	No	VC 54%	–	Sainio et al., 2019 [10]
10	E.II.2	c.464A > G p.(Asn155Ser) c.1159-1160insCAAA	Childhood	Proximal, distal, upper and lower limbs, facial weakness	Internal nuclei, cores	Yes	No	Normal	High-arched palate	O’Grady et al., 2016 [6]
11	A.II.1	c.285 + 1G > A c.1116G > C, p.Gln372His	Childhood	Proximal, distal, axial, upper and lower limbs, facial weakness	Internal nuclei, cores, myofibrillar inclusions, sarcomeric disorganization, rods	Yes	Yes	Abnormal	Joint hypermobility, contractures, rigid spine, pectus excavatum, high-arched palate, dental malocclusion, pes cavus	O’Grady et al., 2016 [6]
	A.II.2		Childhood	Proximal, distal, axial, upper and lower limbs, facial weakness	NA	Yes	No	Normal	Joint hypermobility, rigid spine, high-arched palate, dental malocclusion	
12	P3	c.415-976A > G c.1116G > C, p.Gln372His	Neonatal	Proximal, distal, axial, upper and lower limbs, wheelchair-bound since age 13	Internal nuclei, cores, myofibrillar inclusions, rods	Yes	Yes	NIV since age 15	High-arched feet, hand length asymmetry, low-set ears, decreased bone density	This study

*NIV* non-invasive ventilation, *VC* vital capacity

**Fig. 1:**
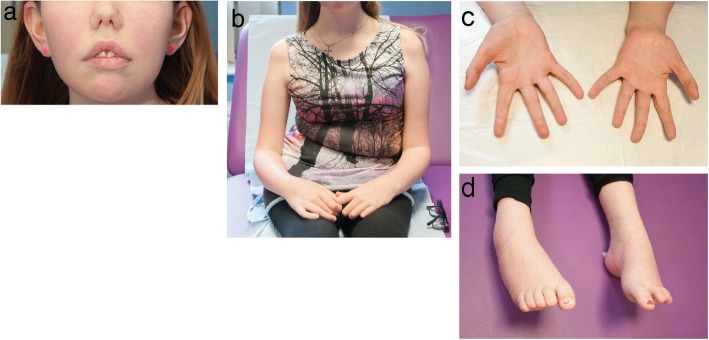
Photographs of P3. **a** Low-set ears, **b** scoliosis, **c** hand size asymmetry, **d** arched feet

P1 and P3 were born to non-consanguineous parents, while the parents of P2 were first-degree cousins. P1 manifested neonatal hypotonia and delayed motor milestones with progressive axial muscle weakness. The patient is wheelchair-bound since the age of 12 years, and respiratory insufficiency requires non-invasive ventilation (NIV) and oxygen therapy since the age of 14 years. Additional clinical features included scoliosis and joint hypermobility. His younger brother was reported with a similar course of disease and perished at the age of 16 years from respiratory distress. Patient 2 had a childhood-disease onset with walking and running difficulties resulting from axial and proximal muscle weakness predominantly affecting the lower limbs. The patient was ambulant at the last clinical examination at the age of 66, and presented with a reduced vital capacity (VC) of 68%. P3 had a similar disease course as P1 with neonatal hypotonia and delayed motor milestones, and a progressive axial, proximal, and distal muscle weakness requiring a wheelchair since the age of 13. Respiratory insufficiency necessitates non-invasive ventilation since the age of 15, and nasal speech, low-set ears, high-arched feet, hand length asymmetry (Fig. 1), mild septal and decreased antero-septal dyskinesia, and reduced bone density were also diagnosed.

Whole body MRI was performed for P1 and P3 and revealed a similar picture with generalized and symmetric atrophy and diffuse fatty infiltrations with particular involvement of proximal lower limb muscles such as gluteus maximus, vastus lateralis, vastus intermedius, and vastus medius.

### Muscle sections show common findings of multiple internalized nuclei and cores

**Fig. 2:**
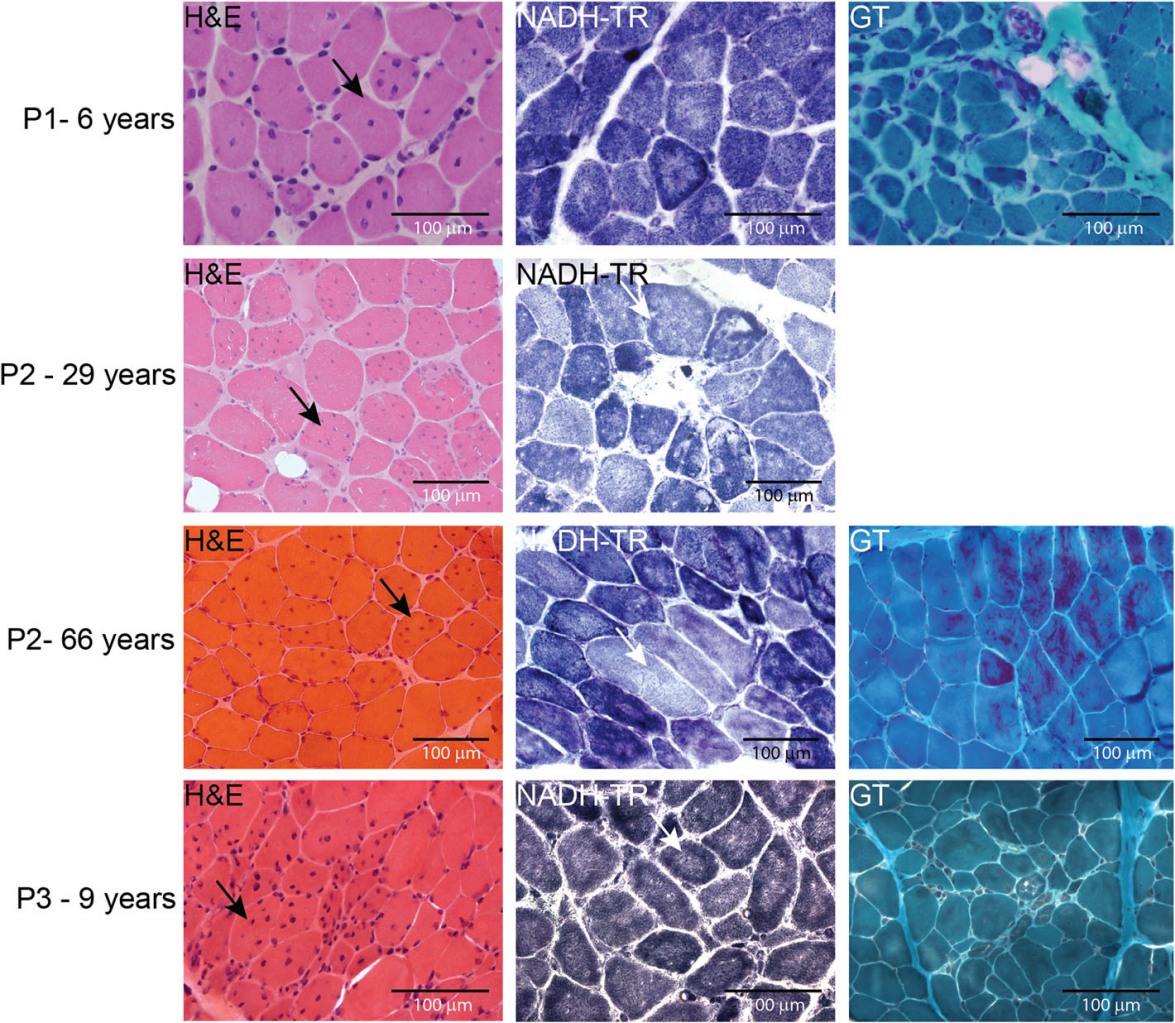
Skeletal muscle histopathology. H&E, NADH-TR, and Gomori trichrome staining of transverse muscle section from P1, P2, and P3 revealed similar histological features as fiber-size heterogeneity, fibrosis, rods, and fibers with multiple internalized nuclei (black arrows) and cores (white arrows)

**Fig. 3:**
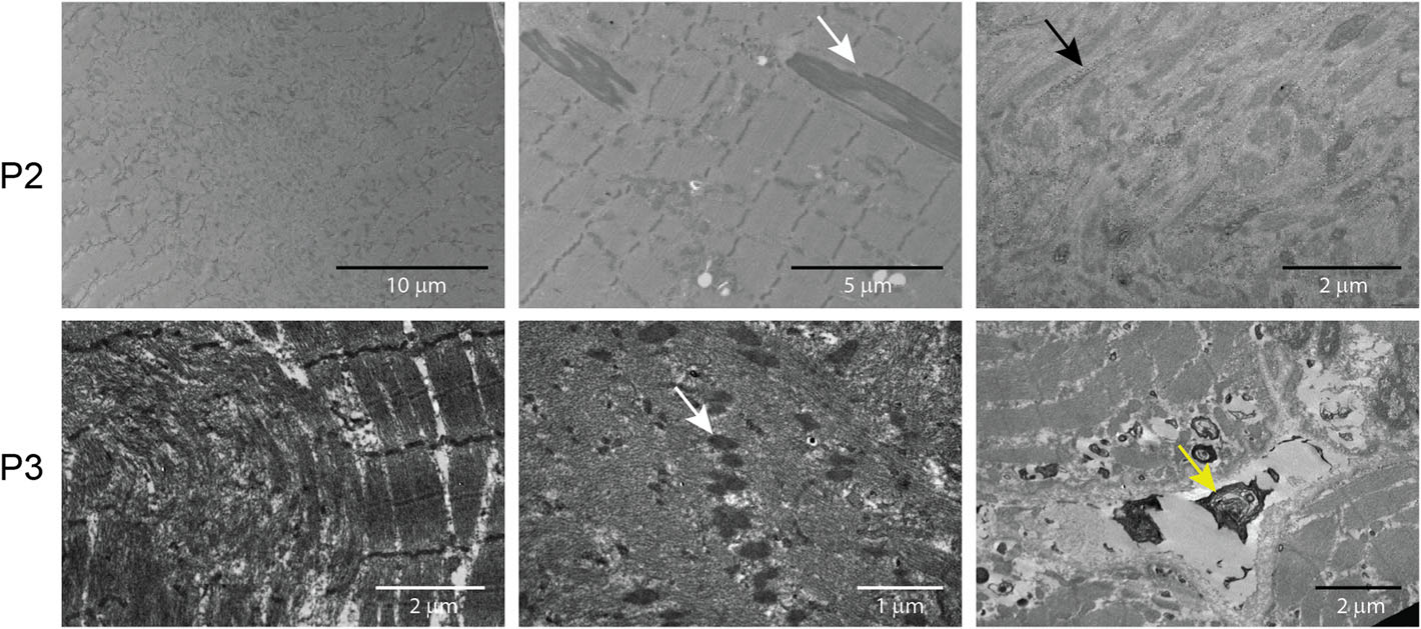
Skeletal muscle ultrastructure. Electron microscopy on muscle section from P2 and P3 confirmed the presence of cores and rods (white arrows), and revealed glycogen accumulations (black arrow), abnormal mitochondria, and dense osmiophilic bodies (yellow arrow) of unknown origin outside the sarcolemma and within fibres

Histological and histochemical analyses on muscle sections from all three patients described in this study revealed fiber size variability, endomysial fibrosis, and especially grouped fibers with multiple internalized nuclei and numerous cores (Fig. 2). Fuchsinophilic inclusions consistent with cytoplasmic rods were furthermore observed on the biopsy from P2 and P3. Ultrastructural investigations on muscle biopsies from P2 and P3 confirmed the presence of cores, rods, and internal nuclei, and uncovered extensive myofibrillar disorganization (Fig. 3). In addition, osmiophilic membranous structures of unknown origin were seen adjacent to the sarcolemma and within fibers in P3.

**Fig. 4:**
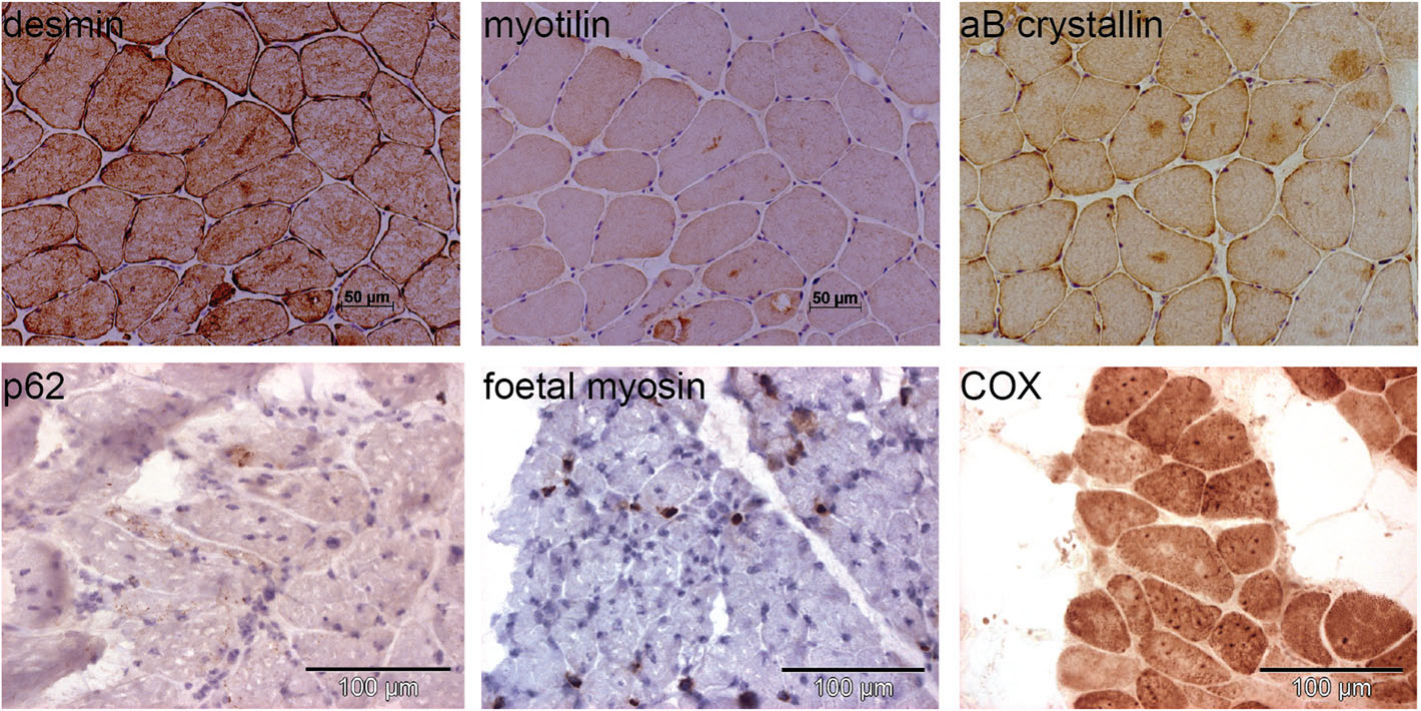
Protein accumulations in patient muscles. Immuno- and chemical staining of muscle biopsies from P2 and P3 revealed accumulations of the myofibrillar proteins desmin, myotilin, and alpha B crystallin, and of the mitochondrial marker COX, and detected a few fibers expressing foetal myosin, or with positive labelling for the p62 autophagy marker

To further characterize the structural aberrations in the muscle fibers, we performed a series of immunolabeling experiments on muscle sections from P2 and P3. We found abnormal aggregations of the intermediate filament desmin, the myofibrillar protein myotilin, and the chaperone alpha-crystallin B (Fig. 4), all three mutated in myofibrillar myopathies. We furthermore detected a subset of fibers expressing foetal myosin, and we noted marked accumulations of the autophagosome marker p62. We also found fibers with dark focal areas strongly staining positive for COX, and areas with reduced COX staining, potentially corresponding to cores. Overall, the clinical, histological, and ultrastructural features of our patients were strongly suggestive of *PYROXD1*-related myopathy.

### Identification of *PYROXD1* mutations

**Fig. 5:**
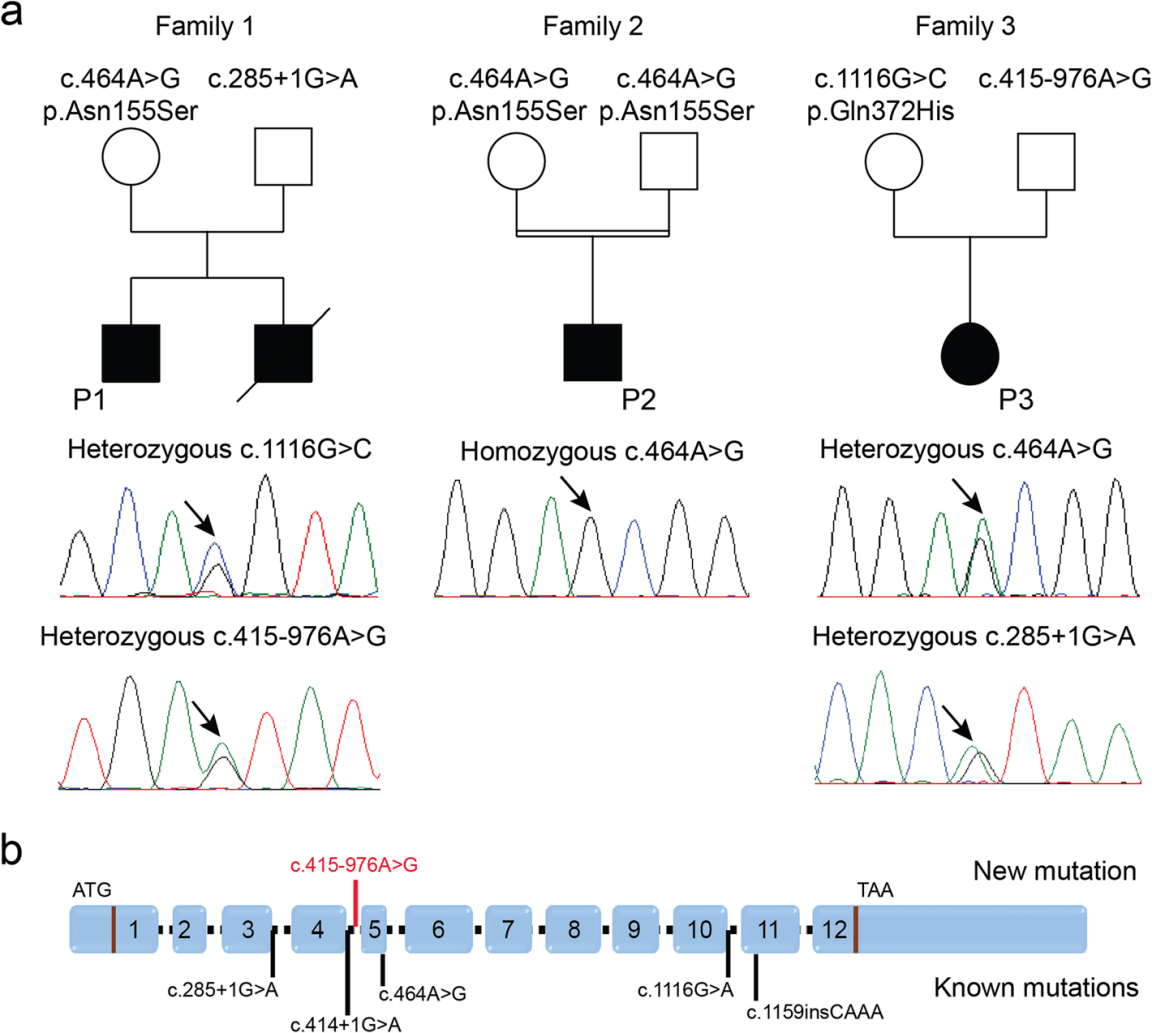
Identification of *PYROXD1* mutations. **a** Pedigrees of three novel *PYROXD1* families and chromatopherograms showing the mutations. **b** Schematic representation of *PYROXD1* and position of known mutations (black) and the novel mutation (red)

**Fig. 6:**
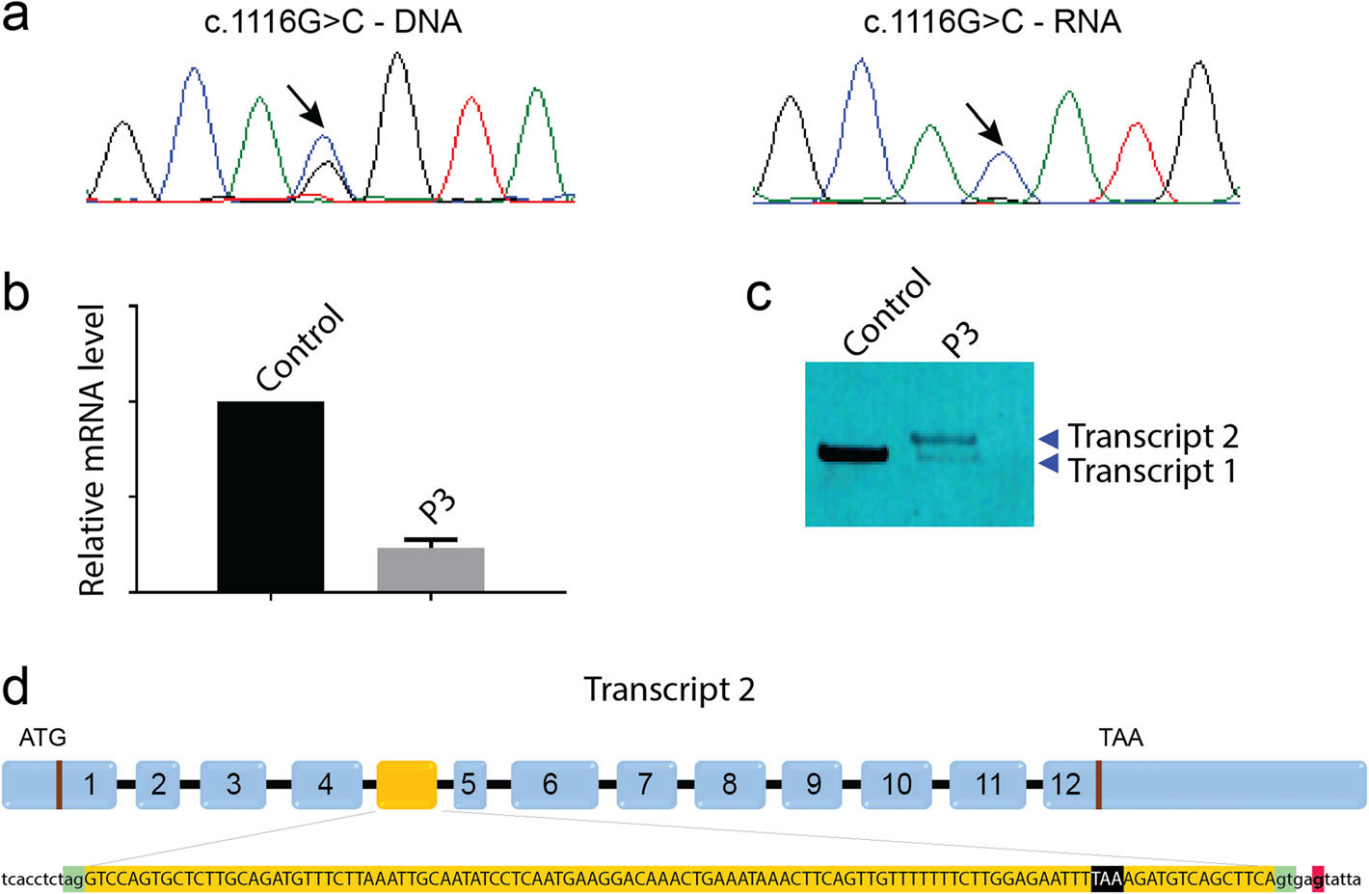
Characterization of the deep intronic mutation. **a** The c.1116G > C mutation appears heterozygous on the P3 DNA and homozygous on the RNA. **b** The *PYROXD1* mRNA was strongly reduced in the muscle from P3. **c** Discriminative PCR on skeletal muscle cDNA revealed the presence of an aberrant amplicon with increased size (transcript 2). **d** Sequencing of the aberrant transcript 2 showed the inclusion of an additional 110 nt exon with in-frame stop codon (highlighted in black). The intronic mutation (red) reinforces a cryptic donor site (green)

We performed panel sequencing of 210 neuromuscular disorder genes for P1 (Family 1), and detected compound heterozygous *PYROXD1* mutations. Segregation analysis disclosed the c.285 + 1G > A mutation affecting the essential donor splice site of the in-frame exon 3 on the paternal allele, and the common *PYROXD1* c.464A > G (p.(Asn155Ser)) missense mutation on the maternal allele (Fig. 5). The younger brother was found to carry the same compound heterozygous *PYROXD1* mutations, confirming recessive disease inheritance. Exome sequencing of P2 revealed the common c.464A > G (p.(Asn155Ser)) missense mutation at the homozygous state, and direct Sanger sequencing of *PYROXD1* in P3 identified the heterozygous c.1116G > C (p.Gln372His) mutation, which was previously reported in an unrelated family [6]. Since the clinical and histopathological features of P3 were strongly indicative of *PYROXD1*-related myopathy, we extracted the skeletal muscle RNA, performed quantitative RT-PCR, and analyzed the reverse transcribed *PYROXD1* coding sequence. We found a strong reduction of the *PYROXD1* mRNA level compared with an age-matched control, and we detected the c.1116G > C (p.Gln372His) mutation at the homozygous state on the cDNA, demonstrating that expression of the second allele was strongly attenuated (Fig. 6). To specifically amplify and enrich the second allele containing the wild-type guanine at cDNA position 1116, we performed PCR using a discriminative primer, and subsequent electrophoresis revealed the presence of a band with increased size (Fig. 6). Extraction and sequencing of the aberrant amplicon uncovered an insertion of 110 nucleotides containing an in-frame stop codon between exons 4 and 5. We next Sanger-sequenced the entire intron 4 on genomic DNA from P3 and detected the deep intronic c.415-976A > G mutation. This transition is not listed in the public databases, and in-silico analyses through NNSplice, MaxEntScan, and SpliceSiteFinder-like predict that it significantly enhances the recognition of a cryptic GT donor splice at positions c.415–979 and c.415–980. Taken together, the c.415-976A > G mutation activates an intronic cryptic slice site and induces the exonisation of 110 nucleotides between exons 4 and 5. The presence of an in-frame stop codon within the cryptic exon presumably leads to nonsense-mediated mRNA decay (NMD) of the aberrant transcript. It has indeed been shown that NMD is efficiently triggered if the stop codon is at least 50–55 nucleotides upstream of the last exon-exon junction [4].

### Increased levels of the stress markers HSP70 and glutathione reductase

**Fig. 7:**
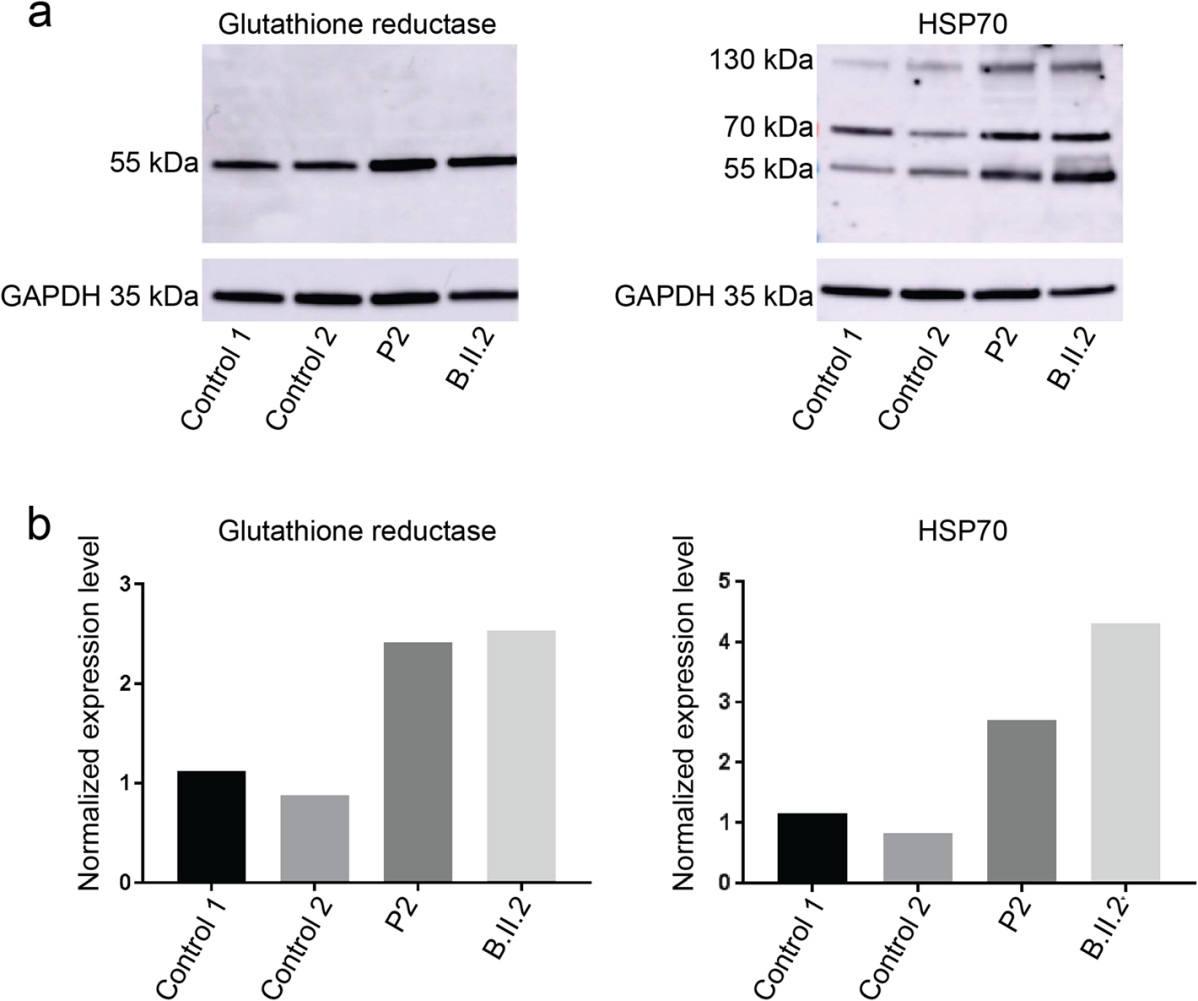
Increased oxidative stress markers. **a** Western blot and **b** quantification on muscle extracts from two *PYROXD1* patients revealed increased protein levels of HSP70 monomers (70 kDa) and dimers (140 kDa) and glutathione reductase compared with age-matched controls

PYROXD1 is a ubiquitously expressed protein containing an oxidoreductase domain, and functional investigations in yeast and mammalian cell models provided the evidence of a reductase activity that can antagonize the effects of oxidative stress [6, 9]. To further investigate the impact of the identified *PYROXD1* mutations on muscle physiology, we assessed the expression levels of HSP70 and glutathione reductase in muscle extracts from P2 and a previously reported PYROXD1 patient with the common p.(Asn155Ser) missense mutation (B.II.2) [6]. HSP70 is a heat shock protein and glutathione reductase is implicated in oxidoreduction, and both are known to be upregulated in skeletal muscle in response to cellular stress [3, 11]. Western blot revealed increased HSP70 and glutathione reductase signal intensities in P2 and B.II.2 compared to age-matched controls, and quantification showed that both proteins are significantly more abundant in the patient muscles (Fig. 7). This suggests that the *PYROXD1* mutations result in increased oxidative stress, which presumably contributes to the skeletal muscle pathology of the patients.

## Discussion

Here we describe three new families with recessive *PYROXD1* mutations, and we support our findings by clinical, histological, ultrastructural, and genetic data. All patients presented with early-onset and progressive myopathy, and the biopsies revealed myofibrillar inclusions and multiple internal nuclei and cores as common features. The abundance of HSP70 and glutathione reductase in the patient muscles suggests that oxidative stress contributes to the pathology.

### Genotype/phenotype correlation

Exome, panel, or Sanger sequencing identified recessive missense or intronic *PYROXD1* mutations in the three patients described in this study. The c.464A > G (p.(Asn155Ser)) missense mutation was found at the compound heterozygous state in P1 and at the homozygous state in P2, and has previously been reported in eight *PYROXD1* families from different ethnic origin [6, 9, 10]. The public gnomAD database lists 12 heterozygous and no homozygous carriers of the *PYROXD1* c.464A > G mutation among almost 140,000 individuals. For other *PYROXD1* mutations as c.285 + 1G > A, p.(Tyr354Cys), and p.Gln372His, gnomAD respectively lists 22, 4, and 5 heterozygous carriers and not a single homozygous carrier, and this low frequency in the general population is in agreement with the rare occurrence of recessive *PYROXD1* mutations associated with a human disorder.

Including our patients, a total of 12 *PYROXD1* families have been described to date, and all had a progressive course of disease with a predominant involvement of upper and lower limb muscles. As a general rule, carriers of homozygous (p.(Asn155Ser)) or compound heterozygous missense mutations (p.(Asn155Ser) and p.(Tyr354Cys)) appear to exhibit a LGMD-like phenotype with childhood or adulthood disease onset and only little additional clinical features (this study and [6, 9, 10]). By contrast, patients with splice mutations (c.285 + 1G > A; c.414 + 1G > A; c.415-976A > G) are most often affected at birth or infancy, and manifest a more severe and complex clinical picture including scoliosis, nasal speech, joint hypermobility, contractures, rigid spine, pectus excavatum, and anomalies of the feet and hands (this study and [6]). It was shown that the mutations affecting the essential splice sites of the in-frame exons 3 and 4 interfere with the production of a stable PYROXD1 protein [6], and here we demonstrated that the deep intronic c.415-976A > G mutation found in P3 impaired transcript expression. This illustrates that all identified splice mutations involve a reduction of the overall PYROXD1 protein level, which manifestly contributes to the development of a more severe and early-onset phenotype. Noteworthy, the splice mutations were exclusively found at the heterozygous state and in combination with a heterozygous missense mutation, suggesting that homozygous splice mutations might be embryonically lethal or give rise to a severe systemic disorders not classified and recognized as myopathies.

### Multiple internal nuclei and cores as histopathological hallmarks

The biopsies from the patients described in this study displayed similar histological features of fiber size variability, endomysial fibrosis, and the presence of characteristic groups of muscle fibers with multiple internalized nuclei and cores. Electron microscopy additionally revealed prominent myofibrillar disorganization and occasional rods, consistent with the histological and ultrastructural observations in previously reported *PYROXD1* cases [6]. Internal and central nuclei, cores of varying size and rods define specific forms of congenital myopathies [8], but a considerable overlap with co-existence of histopathological abnormalities has been described as well [2]. The combination of multiple internal nuclei and cores within single fibers constitutes a typical histopathological indication of *PYROXD1*-related myopathy.

As previously shown [6] and confirmed in the present study, the muscle fibers from *PYROXD1* patients also featured myofiber disorganization and accumulations of myofibrillar proteins as desmin, myotilin, or alpha B crystallin. Although primarily seen in myofibrillar myopathy, the accumulations were also described in different forms of core myopathy [1, 12]. Vacuoles, another hallmark of myofibrillar myopathy, have not been detected in any *PYROXD1* biopsy. Overall and in view of the histological characteristics on biopsies as cores, central nuclei, and sarcoplasmic aggregates, *PYROXD1*-related myopathy can be considered as mixture of core myopathy, centronuclear myopathy, and myofibrillar myopathy. This highlights the relevance of *PYROXD1* sequencing in patients with different histopathological diagnosis.

### Impact of *PYROXD1* mutations on muscle physiology

We investigated the structural muscle fiber aberrations in our patients by additional immunolabeling experiments, and detected foetal myosin and found evidences of enhanced autophagy. PYROXD1 is an oxidoreductase, and complementation assays in the yeast have shown that the missense mutations p.(Asn155Ser) and p.(Gln372His) strongly impair the oxidoreductase activity, and thereby increase the sensitivity of the cells to oxidative stress [6]. Oxidative stress can cause mitochondrial damage and impair mitochondrial function, and was also shown to promote the formation of cores in muscle fibers [7]. As PYROXD1 deficiency was furthermore associated with reduced mitochondrial respiration in cultured myoblasts [9], we may speculate that the aberrant skeletal muscle function and structure in *PYROXD1* patients is partially a consequence of increased oxidative stress. This is supported by our findings of increased expression of HSP70 and glutathione reductase in the patients.

## Conclusions

Nine families with recessive *PYROXD1* mutations have been reported to date, and here we present clinical, histological, and genetic data on three novel families. We expand the genetic spectrum of *PYROXD1*-related myopathy and report for the first time a deep intronic mutation. By comparing all new and published cases, we furthermore provide a genotype/phenotype correlation. *PYROXD1* codes for a barely studied oxidoreductase and should be considered in patients with early-onset muscle weakness predominantly affecting the limbs, especially if the muscle biopsy shows multiple internal nuclei and cores within individual fibers.

## Data Availability

All data generated or analyzed during this study are included in this published article.

## References

[1] Dubowitz Victor, Sewry Caroline A (2007). Normal muscle. Muscle Biopsy.

[2] Gonorazky HD, Bonnemann CG, Dowling JJ (2018). The genetics of congenital myopathies. Handb Clin Neurol.

[3] Ji LL, Fu R (1992). Responses of glutathione system and antioxidant enzymes to exhaustive exercise and hydroperoxide. J Appl Physiol (1985).

[4] Maquat LE (2004). Nonsense-mediated mRNA decay: splicing, translation and mRNP dynamics. Nat Rev Mol Cell Biol.

[5] Nance JR, Dowling JJ, Gibbs EM, Bonnemann CG (2012). Congenital myopathies: an update. Curr Neurol Neurosci Rep.

[6] O'Grady GL, Best HA, Sztal TE, Schartner V, Sanjuan-Vazquez M, Donkervoort S, Abath Neto O, Sutton RB, Ilkovski B, Romero NB (2016). Variants in the oxidoreductase PYROXD1 cause early-onset myopathy with internalized nuclei and Myofibrillar disorganization. Am J Hum Genet.

[7] Paolini C, Quarta M, Wei-LaPierre L, Michelucci A, Nori A, Reggiani C, Dirksen RT, Protasi F (2015). Oxidative stress, mitochondrial damage, and cores in muscle from calsequestrin-1 knockout mice. Skelet Muscle.

[8] Romero NB, Clarke NF (2013). Congenital myopathies. Handb Clin Neurol.

[9] Saha M, Reddy HM, Salih MA, Estrella E, Jones MD, Mitsuhashi S, Cho KA, Suzuki-Hatano S, Rizzo SA, Hamad MH (2018). Impact of PYROXD1 deficiency on cellular respiration and correlations with genetic analyses of limb-girdle muscular dystrophy in Saudi Arabia and Sudan. Physiol Genomics.

[10] Sainio MT, Valipakka S, Rinaldi B, Lapatto H, Paetau A, Ojanen S, Brilhante V, Jokela M, Huovinen S, Auranen M (2019). Recessive PYROXD1 mutations cause adult-onset limb-girdle-type muscular dystrophy. J Neurol.

[11] Salo DC, Donovan CM, Davies KJ (1991). HSP70 and other possible heat shock or oxidative stress proteins are induced in skeletal muscle, heart, and liver during exercise. Free Radic Biol Med.

[12] Selcen D, Shen XM, Brengman J, Li Y, Stans AA, Wieben E, Engel AG (2014). DPAGT1 myasthenia and myopathy: genetic, phenotypic, and expression studies. Neurology.

